# The Ca^2+^ Bridge: From Neurons to Circuits in Rett Syndrome

**DOI:** 10.3390/ijms262110490

**Published:** 2025-10-29

**Authors:** Luis Molina Calistro, Yennyfer Arancibia, Javiera Alarcón, Rodrigo Flavio Torres

**Affiliations:** 1 Facultad de Ciencias, Universidad San Sebastián, Lago Panguipulli 1390, Puerto Montt 5501842, Chile; luis.molina@uss.cl (L.M.C.);; 2 Millennium Nucleus of Neuroepigenetics and Plasticity (EpiNeuro), Santiago 8320000, Chile; 3 Facultad de Medicina, Universidad San Sebastián, Lago Panguipulli 1390, Puerto Montt 5501842, Chile; jalarcono4@correo.uss.cl

**Keywords:** Rett syndrome, Mecp2, calcium, ryanodine receptors, calcium signaling, neuronal function and dysfunction

## Abstract

Rett syndrome (RTT) is a severe neurodevelopmental disorder caused primarily by mutations in the gene encoding the methyl-CpG-binding protein 2 (Mecp2). Mecp2 binds to methylated cytosines, playing a crucial role in chromatin organization and transcriptional regulation. At the neurobiological level, RTT is characterized by dendritic spine dysgenesis and altered excitation–inhibition balance, drawing attention to the mechanisms that scale from mutations in a nuclear protein to altered neuronal connectivity. Although Mecp2 dysfunction disrupts multiple neuronal processes, emerging evidence highlights altered calcium (Ca^2+^) signaling as a central contributor to RTT pathophysiology. This review explores the link between Mecp2 and Ca^2+^ regulation by highlighting how Mecp2 affects Ca^2+^-dependent transcriptional pathways, while Ca^2+^ modulates Mecp2 function by inducing post-translational modifications. We discuss this crosstalk in light of evidence from RTT models, with a particular focus on the brain-derived neurotrophic factor BDNF-miR132-Mecp2 axis and the dysregulation of ryanodine receptors (RyRs). Additionally, we examine how these perturbations contribute to the reduced structural plasticity and the altered activity-driven gene expression that characterizes RTT. Understanding the intersection between Mecp2 function and Ca^2+^ homeostasis will provide critical insights into RTT pathogenesis and potential therapeutic targets aimed at restoring neuronal connectivity.

## 1. Introduction

RTT is a devastating postnatal progressive neurodevelopmental disorder estimated to affect 1:15,000 live births [1,2]. Actually, our current estimation of RTT incidence may be lower than the true value, as some non-syndromic autism cases may in fact be non-diagnosed RTT [3]. RTT manifests in early childhood. However, not all symptoms appear simultaneously. Patients with RTT show normal development until 6–18 months of age when behavioral stagnation becomes apparent. Developmental milestones such as learning to walk or the purposeful use of hands are lost as the disorder progresses and autistic features such as social isolation and loss of language become evident as the patient ages [1,3,4]. Systemic alterations are also associated with RTT. For instance, short statures, weight disorders and precocious puberty and menstrual irregularities are observed in RTT patients [5]. Calling attention to the role played by endocrine hormones, such as estrogen, could help explain the neurobiological characteristics and progression of RTT [6].

Although RTT was first described over 50 years ago, unraveling the basis of such a complex syndrome required developments that favored a better understanding of the processes governing gene expression. One of such processes relies on the chemical modification of DNA. These modifications adjust the chromatin structure and affect the binding capacity of transcription factors, constituting a mechanism through which gene expression can be regulated in a long-lasting manner [7]. Cytosine methylation is a highly dynamic DNA modification with roles ranging from cell differentiation to neuronal function [7,8]. Several proteins containing a methyl-binding domain (MBD) interact with methylcytosines, translating DNA methylation into gene expression regulation. Among these, the first identified and most extensively studied MBD protein is the methylated cytosine binding protein 2 (Mecp2) [9,10,11]. In 1999, the relevance of Mecp2 was highlighted by the discovery that Mecp2 mutations are the main cause of Rett syndrome (RTT, MIM 312750) [10].

The gene *MECP2* is located on the X chromosome and comprises four exons that, through alternate splicing, produce two isoforms that differ in the first exon. These isoforms, namely Mecp2-E1 and Mecp2-E2, are translated into proteins containing 498 and 486 amino acids, respectively. Although Mecp2-E1 shows a slightly larger N-terminal domain (NTD), both isoforms share almost identical amino acid sequences [12]. The observation that Mecp2 isoforms show a differential pattern of expression during early mouse development is indicative of non-redundant functions [12]. However, it has also been shown that the RTT-like phenotype is prevented independently by Mecp2-E1 and Mecp2-E2 transgenes [13], suggesting overlapping functions. The reader can be referred to excellent reviews that will provide a deeper insight into the functional redundancies and discrepancies of Mecp2 isoforms [14,15]. In addition to the common MBD, this protein has a transcriptional repressor domain (TRD) and a carboxy-terminal domain (CTD) [9]. The MBD is necessary and sufficient for methylcytosine binding in a DNA sequence-independent manner. The CTD allows Mecp2-nucleosome and Mecp2-naked DNA interactions [16]. Whereas the TRD has been shown to interact with histone deacetylases (HDACs) among other proteins, therefore compacting the chromatin and silencing gene transcription [9,17]. Although it is tempting to think of DNA methylation and Mecp2 binding as a gene silencing sign, an Mecp2-CREB interaction has also been described [18]. Moreover, Mecp2 also binds to hydroxymethylated cytosines (hmC), particularly in CpA dinucleotides within active genes [19,20], and transgenic mouse models have shown that Mecp2 target genes can be up- or downregulated in the brain [15,21,22], suggesting that Mecp2 function is far more complex than initially thought [15,23].

While there are over 200 different mutations that cause RTT through complete or partial loss of Mecp2 function and accounting for 95–97% of RTT cases, about 60% of typical RTT cases are produced by only eight mutations [4,24,25]. The gene–phenotype relationship is strengthened as the type of Mecp2 mutation is associated with the severity of the disease [24]. This gene–phenotype association is also evident in the several mouse models currently available, ranging from complete Mecp2 deletion to specific mutation-containing Mecp2 variants [4,11,26,27]. The Mecp2-null mice (Mecp2-KO) is the widest used RTT mouse model. It recapitulates the features of RTT, showing a pre-symptomatic window of about 3–4 weeks. After this, several symptoms appear gradually, such as abnormal gait and movements, hypoactivity, hypotonicity, tremor, hindlimb clasping and irregular breathing [26,28,29].

A neurobiological characteristic of RTT is the alteration in neuronal morphology [25,30]. Dendritic complexity and dendritic spine density are reduced in postmortem samples from RTT patients and Mecp2-KO mice [30,31,32,33]. In vivo observation of dendritic spines in Mecp2-KO mice showed that spine dynamics is severely impaired from an early postnatal developmental stage, when RTT-like symptoms are mild [34]. Moreover, Mecp2 deletion in older mice also results in a dramatic reduction in dendritic complexity and dendritic spine density [32], highlighting the role of Mecp2 not only in the formation but also in the maintenance of functional dendritic spines. Dendritic spine dysgenesis has been suggested as a cause for RTT and is considered a relevant therapeutic target [30,35,36]. Hence, understanding the basis of dendritic spine dysgenesis and the mechanisms contributing to unstable connectivity are critical issues in understanding the pathophysiology of RTT.

The reversible nature of RTT has been shown using several approaches. For instance, Mecp2 re-expression in post-symptomatic Mecp2-null mice ameliorates behavioral and functional alterations [37]. Furthermore, the attenuation of RTT characteristics has also been achieved using isoform-specific Mecp2 transgenic complementation in a null background [13]. Recently, Mecp2 re-expression using adeno-associated viral vectors prolonged lifespan and ameliorated behavioral abnormalities associated with RTT in mouse models [38], opening a real possibility for gene therapy to treat RTT. On the other hand, the early manifestation of RTT has occluded the relevance of Mecp2 in aging [39,40]. In this regard, postnatal Mecp2 inactivation revealed a late age window in which a reduction in Mecp2 levels is incompatible with life [41], suggesting that the maintenance of previously established connections is deeply dependent on Mecp2 function. Altogether, such evidence are strong indicators of Mecp2-driven cell-autonomous mechanisms that generate and maintain neuronal connectivity [42].

## 2. Cell-Autonomous Mechanisms and Circuit Consequences

The devastating phenotype associated with RTT emerges from a cell-autonomous perturbation that scales up to disturb neuronal connectivity. For instance, it has been recently reported that functional brain connectivity or neuronal networks, determined as wide field mesoscale calcium measurements, are severely altered in developing and adult Mecp2 null mice [43]. The excitation/inhibition (E/I) balance represents a framework to understand circuit perturbations in the context of neurodevelopmental disorders and other pathologies [30,44]. This framework posits that neuronal alterations drive a reduced signal-to-noise ratio, diminishing the information processing capability of circuits. The signal-to-noise ratio is tightly controlled within neurons and collectively within circuits. For instance, neurons show a regular ratio of excitatory to inhibitory synapses along dendrites, whereas circuits show a regular ratio of excitatory to inhibitory neurons [45,46]. Hence, an increase in excitatory connectivity shifts the E/I balance towards noisier circuits, whereas increased inhibition leads to abnormally silent connectivity. In the context of RTT, the alteration of the E/I balance is observed not only as a perturbation of the signal-to-noise ratio at a circuit level, but also as a functional and morphological alteration of the dendritic spines of individual neurons [25,30,36,47]. This diminishes the available surface area for glutamatergic neurotransmission and drives counterbalancing mechanisms that increase the excitability of glutamatergic neurons, thereby exacerbating the E/I alterations.

Several lines of evidence suggest an altered E/I balance in RTT. For instance, the stimulation of white matter with currents of increasing intensity evoked reduced calcium (Ca^2+^) responses in the medial prefrontal cortex of Mecp2-null mice (mPFC), in contrast to that of WT mice [48,49]. Furthermore, diminished dendritic spine density and reduced abundance of critical excitatory proteins, such as the vesicular transporter of glutamate (vGLUT1), suggests that Mecp2 absence determines a reduction in excitatory connectivity [47,48]. It was further shown that Mecp2 overexpression increases, whereas Mecp2 deletion reduces glutamatergic synapse numbers estimated by vGLUT1, PSD95 and MAP2 colocalization [47,50]. Moreover, a brief postnatal knockdown of Mecp2 was sufficient to reduce the excitatory synapse number by 30% [50]. These findings highlight the relevance of Mecp2 for generating and maintaining glutamatergic connectivity. In the context of chronic changes in network activity, the E/I balance is maintained by the homeostatic adjusting of the synaptic strength, a process known as synaptic scaling. Synaptic scaling is a transcriptional-dependent process, with Mecp2 contributing to scaling-down in the context of elevated network activity, and scaling-up in the context of decreased activity [42,51,52]. For instance, 4 days of visual deprivation increased the amplitude of the miniature excitatory postsynaptic currents (mEPSC) in WT but not in Mecp2-null mice [42], suggesting a critical role for Mecp2 in synaptic scaling-up in vivo. Hence, Mecp2 represents a critical actor to E/I balance, contributing to the regulation of the strength and number of glutamatergic synapses. Collectively, these data position Mecp2 as a nodal regulator, linking cell-autonomous molecular mechanisms to circuit-level perturbations.

The question is, how do cell-autonomous perturbations, such as a Mecp2 mutation, scale up to circuits? The evidence suggests that Ca^2+^ homeostasis is centrally positioned to bridge cell-autonomous perturbations with the widespread connectivity changes associated with the RTT neurobiological characteristics. Ca^2+^ plays a central role in excitation–transcription coupling, contributing to the neuronal adaptation for varying levels of activity associated with brain function. For instance, intracellular calcium transients direct gene expression [53,54,55], post-translational modifications and orchestrate the modification of neuronal architecture [56,57,58]. Furthermore, non-neuronal cell types also show Ca^2+^ disturbances in the context of RTT [59]. Hence, understanding the mechanisms by which Mecp2 drives the alterations in Ca^2+^ signaling observed in RTT should be a primary focus of research as it could open new avenues for pharmacological interventions for this devastating syndrome.

The nature of Mecp2 as a chromatin organizer determines that several genes associated with Ca^2+^ homeostasis could be either direct or indirect Mecp2 downstream target genes (IE genes that are directly affected by Mecp2, either by binding to a promoter or enhancer, or genes whose regulators are targets of Mecp2). However, besides Mecp2 downstream targets, there are also upstream mechanisms associating Ca^2+^ signaling with the post-transcriptional and post-translational regulation of Mecp2. These upstream mechanisms are relevant for coupling neuronal activity with Mecp2 function, contributing to neuronal remodeling in response to activity. In the following sections we will elaborate on both Mecp2 upstream and downstream in relation to Ca^2+^.

## 3. Upstream Mecp2 Post-Translational Regulation by Ca^2+^ Signaling

Mecp2 is subject to several post-translational modifications, including phosphorylation, ubiquitination and SUMOylations [60,61]. Among these, Mecp2 phosphorylation by CaMK II and CaMK IV highlights the importance of Ca^2+^ for the regulation of Mecp2 functions [62]. Neuronal activity drives the brain-specific phosphorylation at serine 421 (pS421) [53,63,64]. On the other hand, neuronal activity reduces the phosphorylation at serine 80 (pS80) [62]. Ca^2+^ entry through L-type voltage-gated Ca^2+^ channel represents a common trigger for the phosphorylation of Mecp2 at S421 and the dephosphorylation of Mecp2-pS80 [62]. Thus, pS421 and pS80 are regulated as mirrored images, directing the dynamic binding of Mecp2 to several gene promoters and affecting the protein partners that interact with Mecp2 under different conditions [62]. Mecp2-pS80 is considered relevant for Mecp2 function in resting neurons, whereas Mecp2-pS421 is deemed as an activity-induced modification [56,62,65]. Both pS421 and pS80 are relevant in directing the association of Mecp2 to chromatin in response to neuronal depolarization, but also relate neuronal activity to the structural remodeling of neurons [65,66].

A major example of Mecp2-pS421 relevance to neuronal morphology and organization comes from the activity-induced transcriptional activity of the brain-derived neurotrophic factor gene (BDNF) [63]. This neurotrophic factor is processed and released upon neuronal activity, contributing to axonal guidance, dendritic arborization and dendritic spine formation and maintenance [67]. BDNF signals through the tropomyosin receptor tyrosine kinase B (TrkB), which recruits the phospholipase C gamma (PLCγ) to promote the formation of IP3 and the concomitant release of Ca^2+^ from intracellular stores.

Neuronal stimulation with BDNF and other neurotrophins increases the level of Mecp2-pS421. In turn, Mecp2-pS421 is required to release Mecp2 from the BDNF promoter and increase its transcription, contributing to the activity-induced upregulation of BDNF [68]. Replacement of Mecp2 for an S421A mutant (Mecp2-S421A) blunted the depolarization -induced transcriptional activation of the BDNF gene [68]. Interestingly, the Mecp2-S421A mutant also showed impaired dendritic spine morphogenesis and altered dendritic patterning in vitro [63,66,68]. In vivo studies, using an S421A knock in mouse, showed that the loss of Mecp2 pS421 increased the locomotor activity in a running wheel and disrupted the behavioral response to novel objects [62,69]. On the other hand, an S80A knock in mouse model showed decreased locomotor activity, resembling the RTT-associated phenotype observed in Mecp2-null mice [62]. These observations highlight the relevance of the Ca^2+^-dependent post-translational regulation of Mecp2 to turn activity-driven transcription into functional circuits. Importantly, sensory stimuli drive Mecp2 phosphorylation, representing a cornerstone in the role of the environmental contribution to brain circuit refinement during development, a period where RTT-associated disturbances arise [62,64].

## 4. Upstream Mecp2 Post-Transcriptional Regulation by Ca^2+^ Signaling

Neurons show a remarkably high level of Mecp2 expression, reaching up to one Mecp2 molecule every two nucleosomes [70]. Notwithstanding, Mecp2 levels are tightly controlled. As a sign of this, even a small increase in Mecp2 expression originates neuronal abnormalities leading to a progressive neurological disorder that shares characteristics with RTT [71]. Hence, understanding how Mecp2 levels are regulated as a function of neuronal activity is relevant to determine RTT pathophysiology.

The micro-RNA 132 (miR132) is a highly conserved micro-RNA that was found in a search for targets of the cAMP response element binding protein (CREB) [72,73]. The miR132 gene shows several functional cAMP response elements (CREs) that link the Ca^2+^ dependent CREB phosphorylation to miR132 transcription [58,72,74]. Several studies have shown that miRNA132 is rapidly induced by depolarization as well as BDNF and CREB signaling [72,74]. In neurons, miR132 is required for activity-dependent dendritic growth [72,75]. This effect is mediated by the translational suppression of p250GAP, a brain-enriched GTPase-activating protein for the Rho family of GTPases [58,74], which determines the increased activity of the Rac1-Pak actin-remodeling pathway [58]. Thus, miR132 overexpression increases neurite outgrowth in vitro and in vivo [75,76,77]. Furthermore, behavioral alterations are also observed in mouse models that knockdown or overexpress this micro-RNA, highlighting its contribution to neuronal organization and ultimately, behavior [75,76,78].

**Figure 1:**
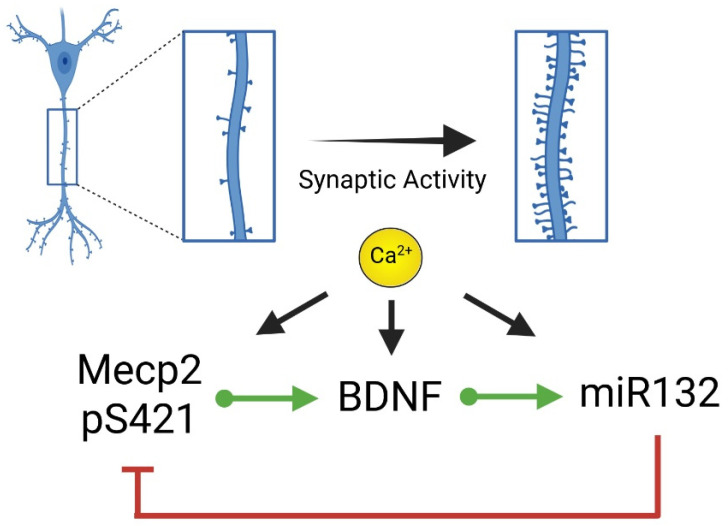
Homeostatic regulation of the BDNF-miR132-Mecp2 axis in synaptogenesis induced by synaptic activity. Synaptic activity drives phosphorylation of Mecp2 at S421, contributing to BDNF increased transcriptional activity. BDNF stimulation upregulates miR132 expression, which in turn suppresses Mecp2. Calcium plays a pivotal role in this axis, orchestrating BDNF and miR132 transcription and Mecp2 phosphorylation. Patients and RTT mouse models show reduced levels of BDNF and miR132, suggesting the contribution of this axis to the reduced structural plasticity associated with this devastating syndrome.

Interestingly, Mecp2 is also a target of miR132, originating a BDNF negative feedback that bridges intracellular modifications to neuronal remodeling [79,80,81]. On one hand, BDNF directs miR132 upregulation and promotes Mecp2-pS421, contributing to activity-dependent BDNF transcription [63,68]. On the other hand, miR132 suppresses Mecp2, providing a homeostatic regulation of BDNF expression (Figure 1) [79,80]. This homeostatic BDNF regulation has been associated with physiological and pathological conditions, including water homeostasis, depression and neurogenesis, among others [80,81,82,83,84,85]. Its relevance becomes apparent when considering that abolishing the miR132-directed suppression of Mecp2 resulted in increased BDNF levels [79,82]. Furthermore, an miR132-KO mouse model showed increased Mecp2 levels and diminished dendritic spine density [83], suggesting its relevance in the RTT-associated phenotype. The reduction in Mecp2 levels by a small dose of siRNA recovered the dendritic spine density, strengthening the association of the miR132-Mecp2 interplay with neuronal morphology [83]. Concordantly, transgenic mice overexpressing the miR132 and mice exposed to an enriched environment show a reduction in Mecp2 levels and increased dendritic spine density [33,75].

The relevance of this homeostatic regulation for RTT is highlighted by the observation that BDNF levels are consistently reduced in RTT patients and mouse models [86,87]. Furthermore, the administration of exogenous BDNF ameliorates RTT-like characteristics in mouse models, positioning BDNF as a central factor in RTT neurobiology [87,88,89]. However, reduced levels of miR132 have also been reported for mouse models and postmortem samples of RTT patients [69,85]. Therefore, it is likely that the loss or alteration of the BDNF-miR132-Mecp2 axis contributes to the reduced structural plasticity and dendritic complexity that characterizes RTT [33,85].

Importantly, Ca^2+^ represents the nodal point for these post-transcriptional and post-translational regulations. For instance, Ca^2+^ entry through NMDA receptors or L-type voltage-gated Ca^2+^ channels drives Mecp2-pS421 and directs BDNF and miR132 transcription [56,57,90,91]. For instance, using organotypic slice cultures and a BDNF reporter, it was shown that the transcriptional response of BDNF to several stimulations was directly associated with the intracellular Ca^2+^ concentration achieved [91]. Furthermore, blocking NMDA and L-type voltage-gated Ca^2+^ channels abolished the transcriptional response of the BDNF promoter [91], underlining the strong association of BDNF transcription with Ca^2+^ signaling. On the other hand, the role of Ca^2+^ in directing the transcriptional activity of miR132 is well described. For instance, the ryanodine receptor (RyR)-mediated calcium release from the endoplasmic reticulum is required for miR132 upregulation, as pharmacological interventions or siRNA downregulation of RyR abolished miR132 upregulation and dendritic remodeling [58,77]. Furthermore, KN62, an inhibitor of Ca^2+^/calmodulin dependent kinase II, impaired the miR132 upregulation induced by neuronal activity [58]. Altogether, activity-induced Ca^2+^ transients orchestrate the BDNF-miR132-Mecp2 axis, translating neuronal activity into dendritic spine remodeling. Hence, Ca^2+^ signaling acts as an upstream bridge from neuronal activity to regulate Mecp2 levels and function.

## 5. Downstream Regulation of Ca^2+^ Signaling by Mecp2

The high levels of Mecp2 expression and its role as a chromatin organizer suggested that Mecp2 deletion or overexpression would result in a vast number of differentially expressed genes. However, the initial findings from the transcriptomics analyses were in contrast with this expectation [21]. The reason behind this discrepancy was later termed as the dilution effect [92]. The dilution effect arises from the specific role Mecp2 plays in different cell types and brain regions. Hence, upregulated genes in a cell type can be downregulated in the neighboring cell. Similarly, diverse brain regions or activity levels within a brain region can lead to opposing transcriptional signatures, masking the results obtained from massive analyses such as transcriptomics. Hence, such studies should be analyzed while paying particular attention to details such as cell types, brain regions and stimulation protocols.

**Figure 2:**
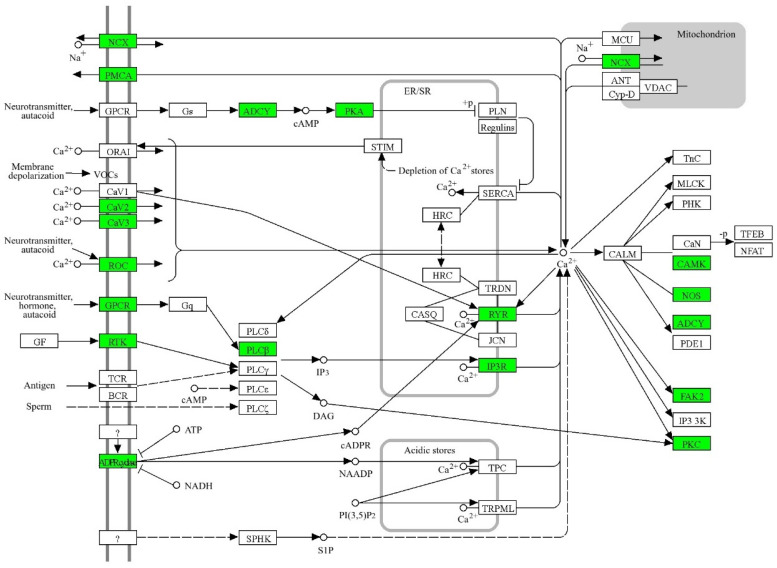
Altered expression of genes associated with Ca^2+^ homeostasis in RTT. The KEGG Ca^2+^ signaling pathway is depicted (ko04020). The genes highlighted in green were found altered according to the meta-transcriptomic analysis of RTT models by Sanfeliu and collaborators, 2019 [93]. Importantly, genes contributing to Ca^2+^ rise in response to depolarization and genes responsible for the intracellular Ca^2+^ liberation from the endoplasmic reticulum are part of the altered pool of genes common to the 38 datasets of RTT models.

A recent effort to determine common pathways altered in RTT emphasized the top genes showing significant differential expression across 38 datasets from transcriptomic studies [93]. Ca^2+^ signaling was ranked the fourth-top KEGG pathway associated with RTT [93]. To draw attention to which Ca^2+^-associated genes are altered in RTT and the possible contribution these genes could have toward RTT pathophysiology, we graphed the Ca^2+^ signaling KEGG pathway and highlighted those genes showing altered levels (Figure 2). Although the nature of the transcriptomic studies does not support these genes as direct Mecp2 targets (IE. direct promoter or enhancer binding), these genes certainly deserve a closer examination, in order to understand their potential contribution to RTT pathophysiology.

Evidence for the direct role played by Mecp2 in the transcriptional regulation of genes associated with Ca^2+^ signaling is sparse, as most efforts have turned into genome wide analyses. However, early and recent transcriptomic studies showed altered expression of RyR2 and RyR3 in Mecp2-null brain tissue [21,93]. RyRs are relevant to synaptic function as they are critical mediators of Ca^2+^-induced Ca^2+^ release (CICR) from the endoplasmic reticulum, contributing to Ca^2+^ propagation from the dendritic spine to the nucleus [77,94,95]. Hence, RyRs participate in neuronal function at several levels, including neuronal remodeling in response to BDNF, long term potentiation and excitation–transcription coupling [55,96,97].

Behavioral stimulation by exposition to an enriched environment or training in the water maze increases RyR3 expression [33,96]. Importantly, these stimulations increase promoter methylation and direct Mecp2 binding [33,98]. On the other hand, RyR3 downregulation was associated with increased promoter hydroxymethylation [33,98]. This strengthens the association between DNA modifications and the transcriptional regulation of these Ca^2+^ channels. Furthermore, Mecp2-null mice were unable to increase the transcriptional activity of RyR2 and RyR3 despite 5 weeks of continuous exposure to an enriched environment, underlining the relevance of Mecp2 in directing the upregulation of RyR transcriptional activity. Importantly, RyRs are also associated with the Mecp2-BDNF-miR132 axis. For instance, WT mice exposed to an enriched environment showed the miR132-depedent p250GAP and Mecp2 suppression. Consequently, these WT mice showed an increase in hippocampal dendritic spine density. On the other hand, Mecp2-null mice failed to show p250GAP suppression and hippocampal dendritic spine remodeling [33], highlighting the relevance of RyRs for activity-dependent neuronal remodeling and suggesting that reduced RyR expression contributes to the altered neuronal morphology associated with RTT [33,77].

RyRs have also been associated with the regulation of autophagic flux. The autophagic flux is essential for maintaining neuronal function by promoting the recycling of cellular components and the elimination of altered organelles. For instance, it was shown that RyR inhibition decreases autophagic flux in neurons [99], and this loss of neuronal autophagy increases presynaptic probability of release by axonal RyR accumulation and subsequent RyR-mediated calcium release, associating RyRs to autophagy and neuronal function [100]. In this line, it was recently reported that Mecp2-null neurons show reduced autophagy and that the pharmacologically induced restoration of autophagy recovers neuronal activity and morphology [101]. Hence, it is possible that reduced RyR expression in Mecp2-null neurons contributes to the impaired autophagy observed in these cells, leading to the abnormal accumulation of cellular components and to a progressive alteration in synaptic plasticity, which is an issue that could be further studied in the context of RTT pathophysiology. Altogether, RyRs are critical mediators of neuronal function through CICR and have been directly associated with Mecp2. Furthermore, RyR expression is altered in pre-symptomatic Mecp2-null mice, raising the need for a deeper understanding of its role in RTT pathophysiology.

It is also relevant to assess the possible contribution of endocrine hormones to the neurobiological characteristics of RTT. The precocious puberty and altered menstrual cycle factors observed in RTT suggests hormonal disturbances that could be associated with the neurobiological characteristics of RTT [5]. For instance, estradiol (E2), is relevant during brain formation, acting as a trophic and neuroprotective factor [102,103]. Furthermore, E2 fluctuations may contribute to the severity and progression of neurodevelopmental disorders [6,103]. E2 acts through its classical nuclear estrogen receptors α and β (ERα and ERβ). Importantly, Mecp2 has been associated with the developmental regulation of ERα expression in the mouse cortex. Concordantly, mice harboring a truncated Mecp2 gene showed an altered pattern of expression of this estrogen receptor [104]. However, apart from the classical estrogen receptor, the G-coupled estrogen receptor 1 (GPER1) is an atypical, membrane-bound estrogen receptor that mediates non-genomic responses to E2. GPER1 signals through mitogen activated kinase (MAPK) and phosphoinositide 3-kinase, (PI3k) driving an intracellular Ca^2+^ rise associated with phospholipase C activity and concomitant inositol triphosphate signaling (IP3). GPER1 has been detected in association to the postsynaptic protein 95 (PSD95) contributing to hippocampal synaptic plasticity [105,106]. Furthermore, GPER1 activity has been associated with learning, memory and social behaviors [107]. Interestingly, GPER1 has been suggested as a target of interest for neurological disorders, and in recent transcriptomic studies, reduced GPER1 expression has been observed in the blood and brains of Mecp2 null mice, opening the possibility that Mecp2 acts as a transcriptional regulator of this receptor [108,109]. Furthermore, it has been reported that E2 potentiates L-Type Ca^2+^channels in neurons directly and by a non-classical membrane estrogen receptor such as GPER1 [110,111], which opens the possibility that endocrine alterations could directly contribute to the Ca^2+^ signaling alterations associated with RTT.

Recently, a meta-analysis of the transcriptomic data from a mouse brain, postmortem human brain tissue and iPSC-derived neurons called attention to the alpha-1A subunit of the voltage-dependent Ca^2+^ channel gene (CACNA1A) [112]. This gene is predominantly expressed in the central nervous system and has been associated with several neurological conditions including epileptic encephalopathy, cerebellar ataxia and developmental disorders [113]. This meta-analysis found CACNA1A to be a hub spot downregulated across these RTT models [112]. Interestingly, a report on a female patient diagnosed with atypical RTT showed a period of behavioral regression, loss of the purposeful use of hands, stereotypic hand movements and impaired gait in absence of MECP2, CDKL5 or FOXG1 mutations, and was subject to genetic studies. DNA sequencing revealed a de novo point mutation in the *CACNA1A* gene [114]. Although no functional assays have been reported, this mutation together with transcriptomic studies suggests a direct association of the *CACNA1A* calcium channel gene with the emergence of the RTT phenotype, suggesting its consideration once mutations associated with classical RTT have been discarded. Strengthening the association of the CACNA1A and RTT phenotype will open a new pathway to understanding RTT physiopathology.

The Transient Receptor Potential Vanilloid 1 (TRPV1) is a ligand and thermosensitive cation channel located mainly in primary nociceptive neurons. At negative potentials, TRPV1 opening causes Na^+^ and Ca^2+^ influx, resulting in cell depolarization and the initiation of nociception [115]. For instance, WT mice injected via the tongue with complete Freund’s adjuvant (CFA) showed increased heat sensitivity accompanied by increased TRPV1 immunoreactivity in the trigeminal ganglion neurons. However, the hyperalgesia induced by CFA injection was not observed in Mecp2^−/+^ heterozygous female mice. Concordantly, TRPV1 immunoreactivity remained unaltered, suggesting the involvement of Mecp2 in heat sensitivity and increased TRPV expression in this model of hyperalgesia [116]. Additionally, a TRPV1 agonist, piperine, showed potential in rescuing some phenotypic alterations associated with RTT. For instance, piperine reduced RTT-associated breathing pauses and increased the locomotor activity in Mecp2-KO mice, suggesting a role for the TRPV1 cation channel in RTT pathophysiology [117]. This phenotypic recovery is better understood by considering that TRPV1 expression extends beyond nociceptive pathways to the central nervous system, including the prefrontal cortex, hypothalamus, cerebellum and hippocampus, among others [118]. Here, TRPV1 expression has been associated with pre- and postsynaptic function, contributing to the presynaptic Ca^2+^ influx required for neurotransmitter release and postsynaptic Ca^2+^ mediated calcineurin activation [118]. This suggests that the phenotypic recovery observed in Mecp2-KO mice could be actively associated with TRPV1-mediated calcium influx.

The relevance of Ca^2+^ signaling as a key mediator of the neuronal alterations associated with RTT has been recently made evident by the use of a novel genetic enhancer of L-type Ca channel, GeeC_L_ [119]. This genetic enhancer increases the open probability of the L-type Ca^2+^ channel, potentiating its function. Interestingly, an engineered human neuronal model of RTT expressing the GeeC_L_ enhancer showed signs of recovery. For instance, CREB phosphorylation was restored, reversing the reduced excitation–transcription coupling associated with RTT neurobiology. Similarly, GeeC_L_ expression in this neuronal model of RTT increased the soma size of neurons, recovering the reduced soma size associated with RTT, paralleling the results obtained by re-expressing Mecp2 from the inactivated X chromosome [120]. Altogether, these observations further enhance the relevance of understanding both the upstream and downstream relations of Mecp2 and Ca^2+^ signaling.

## 6. Conclusions

Years of great-quality research and recent transcriptomic studies have drawn attention to Ca^2+^ signaling as a key contributor to RTT neurobiology. The evidence suggests a central role for Mecp2 in maintaining Ca^2+^ homeostasis, and that perturbations in the Mecp2-Ca^2+^ interplay are direct contributors to synaptic and morphological alterations that characterize RTT. Furthermore, recent evidence suggests that Ca^2+^ signaling potentiation recovers neuronal disturbances associated with RTT. However, there is still a profound need for functional studies of Ca^2+^ signaling genes to assert which targets are specifically Mecp2 targets and most importantly, how they contribute to RTT pathophysiology. Such an effort could help to open pathways for new pharmaceutical targets for this devastating syndrome.

## Data Availability

No new data were created or analyzed in this study. Data sharing is not applicable to this article.
